# The Endocannabinoid System and Cannabidiol's Promise for the Treatment of Substance Use Disorder

**DOI:** 10.3389/fpsyt.2019.00063

**Published:** 2019-02-19

**Authors:** Yann Chye, Erynn Christensen, Nadia Solowij, Murat Yücel

**Affiliations:** ^1^Brain and Mental Health Research Hub, Monash Institute of Cognitive and Clinical Neurosciences, School of Psychological Sciences, Monash University, Melbourne, VIC, Australia; ^2^School of Psychology and Illawarra Health and Medical Research Institute, University of Wollongong, Wollongong, NSW, Australia; ^3^The Australian Centre for Cannabinoid Clinical and Research Excellence, New Lambton Heights, NSW, Australia

**Keywords:** endocannabinoid system, ECS, substance use disorder, treatment efficacy, cannabidiol, CBD, addiction

## Abstract

Substance use disorder is characterized by repeated use of a substance, leading to clinically significant distress, making it a serious public health concern. The endocannabinoid system plays an important role in common neurobiological processes underlying substance use disorder, in particular by mediating the rewarding and motivational effects of substances and substance-related cues. In turn, a number of cannabinoid drugs (e.g., rimonabant, nabiximols) have been suggested for potential pharmacological treatment for substance dependence. Recently, cannabidiol (CBD), a non-psychoactive phytocannabinoid found in the cannabis plant, has also been proposed as a potentially effective treatment for the management of substance use disorder. Animal and human studies suggest that these cannabinoids have the potential to reduce craving and relapse in abstinent substance users, by impairing reconsolidation of drug-reward memory, salience of drug cues, and inhibiting the reward-facilitating effect of drugs. Such functions likely arise through the targeting of the endocannabinoid and serotonergic systems, although the exact mechanism is yet to be elucidated. This article seeks to review the role of the endocannabinoid system in substance use disorder and the proposed pharmacological action supporting cannabinoid drugs' therapeutic potential in addictions, with a focus on CBD. Subsequently, this article will evaluate the underlying evidence for CBD as a potential treatment for substance use disorder, across a range of substances including nicotine, alcohol, psychostimulants, opioids, and cannabis. While early research supports CBD's promise, further investigation and validation of CBD's efficacy, across preclinical and clinical trials will be necessary.

## Introduction

Substance use disorder (SUD) is a global problem, with over 30 million individuals estimated to have an SUD (1). Within the United States alone, SUD-related expenditure (e.g., treatment and productivity cost) exceeded 23 billion USD per year (2), presenting a worrisome issue. Treatment to date has had minimal success, with a high likelihood of relapse (3). There is also no reliably established pharmacotherapy for SUDs, such as cannabis, and stimulant use disorder; and current pharmacotherapies (e.g., opiate substitution with methadone; naltrexone for alcohol use disorder; nicotine replacement) have limited efficacy in relapse prevention (4, 5). SUD has been conceptualized as a maladaptive and relapsing cycle of intoxication, binging, withdrawal and craving that results in excessive substance use despite adverse consequences (6). Recent models implicate major brain circuits involved in reward saliency, motivation, and memory/learned associations in maintaining addiction (7). Critically, these circuits may largely be modulated by the endocannabinoid system (ECS), presenting a promising pharmaceutical avenue for treating SUDs.

## The Endocannabinoid System

The ECS consists of cannabinoid receptors (e.g., CB1R, CB2R), the endogenous ligands that bind to these cannabinoid receptors [e.g., anandamide and 2-arachidonoylglycerol (2-AG)], and enzymes for their biosynthesis and degradation [e.g., fatty acid amide hydrolase (FAAH) and monoacylglyrecol lipase (MAGL)] (8). Over the past decade, primary interest has focused on CB1Rs, for their purported role across a range of physiological functions, including directing the psychoactive effect of delta9-tetrahydrocannabinol (THC), a phytocannabinoid present in cannabis (8, 9). CB1Rs are one of the most common G-protein-coupled receptors in the central nervous system, preferentially residing on presynaptic neurons across diverse regions including the neocortex, striatum, and hippocampus (10, 11). Their widespread distribution allows them to guide a host of functions ranging from cognition, memory, mood, appetite, and sensory responses (8). Endocannabinoids themselves function as neuromodulators that are released by post-synaptic neurons, and bind to the presynaptic CB1Rs to moderate the release of neurotransmitters, such as gamma-aminobutyric-acid (GABA), glutamate, and dopamine (DA) (10, 12, 13). While the specific CB1R function depends on the cell population and region in which they reside, their role in retrograde signaling permits them to regulate signaling activity across cognitive, emotive, and sensory functions, lending therapeutic capacity (14).

## ECS Role in Reward Signaling

Of the functions that the ECS is involved in, of critical interest, is its influence on the brain reward circuitry, particularly in response to substances of abuse. The rewarding effect of substances of abuse is thought to be primarily mediated by the mesolimbic DA pathway, originating from dopaminergic cell bodies in ventral midbrain [ventral tegmental area (VTA)], carrying reward-related information to the ventral striatum [nucleus accumbens (NAc)] (15). The acute reinforcing effect of addictive substances is thought to be due to their direct or indirect activation of DA neurons along this pathway (16). The VTA-NAc pathway as such plays a key function in reward assessment, anticipation, and valuation, making it a critical component underlying substance use and addiction (17).

DA activity is intrinsically tied to cannabinoid activity. CB1Rs are particularly densely located across the striatal regions that mediate reward function (i.e., NAc and VTA) (18), and their regulatory role on the VTA-NAc pathway may be crucial in modulating overall reward tone (19, 20). Rodent studies have demonstrated that THC increases neuronal firing rates in the VTA (21), likely through local disinhibition of DA-ergic neurons, by binding to CB1Rs present on glutamatergic and/or GABAergic neurons (although it is prudent to note that THC's capacity to potentiate DAergic release differs between rodents and humans) (15, 20, 22, 23). Similarly, other substances of abuse (e.g., opioids, cocaine) have also been demonstrated to potentiate dopaminergic activity via the ECS (24, 25). For example, alcohol is found to have a downstream potentiation effect on the ECS in rats (26), such as an increase in endogenous cannabinoid (anandamide and 2-AG) levels (27, 28) and downregulation of CB1R expression (29). Alcohol-induced DAergic release is furthermore dependent on the presence of CB1Rs (30). Nicotine activates DA neurons in the VTA either directly through stimulation of nicotinic cholinergic receptors or indirectly through glutaminergic nerve terminals that are modulated by the ECS (31). Meanwhile opioid receptors are often co-located with CB1Rs in the striatum (32), and may be modulated by and interact with CB1R activity reciprocally (33, 34). Only psychostimulants are suggested to act directly on DAergic axon terminals in the NAc, potentially avoiding upstream endocannabinoid involvement in the VTA (35).

CB1R's role in the motivational and reinforcing effects of rewards has been demonstrated in animal models with CB1R agonists. For example, acute exposure to CB1R agonists (e.g., THC; CP 55,940; WIN 55,212-2; HU 210) augments NAc DA transmission (36), lowers the brain-reward threshold (17), induces conditioned place preference (CPP) (37), and establishes persistent self-administration of substances of abuse, including cannabis and alcohol (17, 38). Meanwhile, CB1R antagonists (e.g., rimonabant) have been shown to attenuate reinforcing effects of these substances, blocking the increase of DA release in the NAc (37, 39). While substances of abuse, such as alcohol, stimulants, nicotine and opioids have differing upstream mechanisms of action (14, 40), the evidence suggest the downstream involvement of the ECS in their reward mechanism.

In summary, the ECS, by direct CB1R activity, modulates and is modulated by mesolimbic DA activity (41). While the action of individual substances may differ, they share a common effect of precipitating DAergic activity from the VTA neurons (42), with this DA-ergic activity mediated by the ECS (14). It is thus thought that the disruption of endocannabinoid signaling may prove effective in treating SUDs (41). Nevertheless, it is necessary to note that this is a simplistic understanding, given the potential involvement of non-DA-ergic neurons in the VTA, and additional neuronal circuits including those involving glutamatergic and opioids, that are yet to be fully elucidated (39, 43).

## ECS Role in Substance Use Disorder (SUD)

Besides the ECS role in reward, it is necessary to acknowledge that substance reward and reinforcement are different from substance dependence. Where the former explain initial substance use, and are suggested to be related to increased DA in striatal and limbic (NAc and amygdala) regions (44, 45); the latter reflects further compulsive substance intake, loss of control, and persistent intake despite the substance's adverse effects and tolerance to its pleasurable responses (44, 46, 47).

Several lines of thought suggest SUD to be a learned habit (48, 49) mediated by persistent changes in striatal function (e.g., synaptic plasticity occurring during learning) (50). Substances of abuse are thought to influence long-lasting plastic changes across corticostriatal circuits, through repeated perturbation of DA activity, thus making it difficult for addicts to cease their substance use, and enhancing risk of relapse (48, 50–52). In this role, CB1Rs present across the corticostriatal circuits, such as the PFC and striatum, mediate synaptic transmission, in their capacity as neuromodulators (35, 53). Evidence demonstrates the necessity of cannabinoid signaling on CB1Rs to induce long-lasting synaptic plasticity, such as long-term depression (LTD) of glutamatergic release across the dorsal and ventral striatum (19, 54). Such functional changes, particularly across the striatal structures responsible for the rewarding and motivational effects of substances of abuse, are not only necessary in providing reward salience, but also in establishing compulsive substance use habit (39, 55). The ECS thus represents a necessary contributor toward cellular adaptations in the transition from recreational substance use to a use disorder (50, 56).

A further function of ECS-mediated synaptic plasticity may be to facilitate emotional learning and memory processes, which promote increased emotional response to substance-related cues (57). The limbic system, in particular the amygdala and hippocampus, by supporting the formation of associative memory, promotes positive and negative reinforcement of rewards including those of substances of abuse (58). Indeed, animal models demonstrate memory performance to not only be dependent on emotional processes, but may be modulated by augmentation of ECS signaling (59–62). Phytocannabinoids, such as THC and CBD for example have been found to modulate brain activity level across limbic regions during emotional processing tasks (63, 64). Endocannabinoids may further induce long-term changes in synaptic strength across the hippocampus, mediating associative memory formation (65–67). Literature investigating cannabinoid agonists and antagonists on SUD solidifies the role of the ECS in emotional learning and memory processes. CB1R agonists and antagonists have respectively been demonstrated to facilitate and attenuate memory extinction in various fear and reward conditioning paradigms in animal models [see (57) for review]. Within the context of SUD, cannabinoid modulation of emotional memory may have implications for extinction, consolidation, and reinstatement of substance-related memory (68). These processes are primarily assessed through place conditioning paradigms, such as CPP. CB1R antagonism by rimonabant for example, has been demonstrated to disrupt the reconsolidation and facilitate the extinction of CPP to substances of abuse, such as methamphetamine and cocaine, potentially via disrupting reward-associated memory (69, 70). Nevertheless, evidence on SUD behavior is mixed and potentially dependent on type and dose of cannabinoids (70, 71).

The ECS's role in reward signaling and learning may as such shape addictive behavior in SUD. The following section details evidence of CB1R's involvement in SUD as demonstrated by cannabinoid agonism and antagonism in animal models.

### Agonism of CB1R

CB1R agonism (either studied with the synthetic cannabinoid agonist WIN 55-212,2 or contrasted against CB1R knockout mice) has been shown to facilitate alcohol self-administration, CPP, and binge-like behavior in animals (38, 72–74). WIN 55,212-2 has also been found to increase motivation to self-administer nicotine, and facilitate cue-induced reinstatement in rats (75). Similar results are found in the heroin literature, with THC-induced CB1R agonism increasing substance self-administration in rats (76, 77).

Agonist substitution with CB1R agonists may have potential for treatment of cannabis use disorder by reducing withdrawal symptoms and the reinforcing effect of cannabis (78). Dronabinol—a stereoisomer of THC, and Nabilone—a synthetic analog of THC, originally intended for nausea and weight loss (55), have both been shown to have efficacy for cannabis withdrawal (79, 80). However, Dronabinol and Nabilone may not prevent cannabis use or relapse (78). It is likely that while these substances are efficacious in attenuating withdrawal symptoms by acting as a “proxy-substances,” they do not directly normalize substance use-related circuits and behavior.

### Antagonism of CB1R

CB1R antagonism has originally been assumed to be a promising target for SUD treatment. SR141716, known as rimonabant, an inverse agonist of CB1R, has been extensively investigated in SUD for its antagonist effect on drug seeking and relapse behavior in both animal and human models.

Animal studies have shown rimonabant as effective in reducing self-administration of alcohol (81, 82), nicotine (83, 84), and heroin (85). Antagonism of CB1R by rimonabant, reduces alcohol-induced sensitization and reinstatement of nicotine-seeking in rats (83, 84, 86). When investigating the efficacy of CB1R antagonists on stimulant use however, the literature is mixed. While rimonabant's CB1R antagonism has been shown to block CPP and attenuate cue- and substance-induced relapse to psychostimulants, such as cocaine and methamphetamines (87–89), evidence pertaining to self-administration is inconsistent (90–92).

Human studies have also been conducted investigating the efficacy of rimonabant in cannabis, nicotine, and alcohol use. Cannabis and nicotine use have both shown sensitivity to rimonabant antagonism. Rimonabant attenuated the acute physiological effects of cannabis including subjective level of intoxication (93, 94), and clinical trials demonstrate rimonabant to be effective in increasing smoking cessation (95). However, the efficacy of rimonabant for alcohol cessation has been less promising. In a 12-weeks clinical trial of relapse rate in recently detoxified alcohol-dependant patients, rimonabant only had a modest effect (that did not reach significance) compared to placebo (96). Rimonabant also had no effect on alcohol consumption for non-treatment seeking heavy alcohol drinkers (97).

Despite promising findings of rimonabant against substance use and relapse, it has been found to produce significant negative psychiatric effects including depression, anxiety, and an elevated suicide rate, preventing it from being a viable treatment option (98). Nevertheless, the evidence indicates CB1R antagonism to have robust effects on some SUDs, highlighting a potential target for SUD treatment. One such candidate drug that antagonizes CB1R, and is increasingly being investigated as a therapeutic option for SUD, is cannabidiol (CBD).

## Cannabidiol (CBD)

CBD is a phytocannabinoid found in cannabis that has recently emerged as a promising treatment for SUDs (99, 100). CBD is non-rewarding, and acts on a number of receptor systems including the opioid (101), serotonergic (102, 103), and cannabinoid (22) systems. Within the cannabinoid system, it is a non-competitive antagonist of CB1R with a low affinity for CB1Rs' primary ligand site (104, 105), instead acting through negative allosteric modulation (105, 106). CBD is found to inhibit endocannabinoid signaling in a dose-dependent manner, likely by binding to CB1Rs' allosteric site and altering the potency of other primary ligands (e.g., endocannabinoids, THC) (106, 107). Its ability to modulate overall ECS tone despite lacking intrinsic efficacy (105) meant that it may decrease CB1R activity without CB1 inverse agonist-related side effects, such as those produced by rimonabant (108, 109). Indeed, CBD has a good safety profile, with generally mild side effects in animal preclinical studies or human studies (110, 111). This, coupled by the limited abuse liability of CBD (112, 113), makes it a good therapeutic candidate. Systemically administered CBD has also been demonstrated to regulate mesolimbic DA activity (114), and potentially attenuate substance-induced dysregulation of the mesolimbic circuitry (115, 116), suggesting its utility against SUDs. Though its efficacy may be dependent on a range of factors including the sequence of administration (i.e., whether CBD is administered in conjunction with, prior to, or post substance-use), and dose ratio (117). A number of papers are urging for the investigation of CBD as a therapeutic option for SUD of multiple substances including stimulants (118), opioids (119, 120), and nicotine use disorder (31). The following section details evidence of CBD treatments for cannabis, alcohol, nicotine, opioid, and stimulants. Table 1 further lists this evidence by SUD constructs.

**Table 1:** CBD's efficacy for the treatment of substance use disorders.

**Study**	**Sample**	**Substance**	Treatment^*^	Outcome^*^	**Effect**
**SENSITIZATION**					
Filev et al. (121)	Mice	Ethanol	CBD (2.5 mg/kg)	Locomotor activity	–
			THC:CBD (2.5:2.5 mg/kg)	Locomotor activity	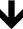
Gerdeman et al. (54)	Rats	Heroin	THC:CBD (10:10 mg/kg)	Locomotor activity	–
Luján et al. (122)	Mice	Cocaine	CBD (20 mg/kg)	Locomotor activity	–
**REWARD FACILITATION**					
Trigo et al. (123)	Humans	Cannabis	THC:CBD (27:25 mg/ml) as needed + MET and CBT	Craving—MCQ	–
Solowij et al. (124)	Humans	Cannabis	Daily oral CBD (200 mg)	CEQ euphoria	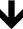
Morgan et al. (125)	Humans	Nicotine	CBD as needed	Craving—TCQ	–
Hindocha et al. (126)	Humans	Nicotine	CBD (800 mg)	Craving—QSU-B	–
			CBD (800 mg)	Attentional bias—visual probe task	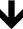
			CBD (800 mg)	Pleasantness rating	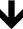
Markos et al. (127)	Mice	Morphine	CBD (2.5 mg/kg)	CPP	–
			CBD (5 mg/kg)	CPP	–
			CBD (10 mg/kg)	CPP	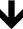
			CBD (20 mg/kg)	CPP	–
Luján et al. (122)	Mice	Cocaine	CBD (5 mg/kg)	CPP	–
			CBD (10 mg/kg)	CPP	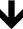
			CBD (20 mg/kg)	CPP	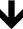
			CBD (30 mg/kg)	CPP	–
Parker et al. (113)	Rats	Amphetamine	CBD (5 mg/kg)	CPP	–
**SELF-ADMINISTRATION**					
Shannon et al. (128)	Human: case study	Cannabis	CBD (24-18 mg)	Abstinence	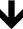
Trigo et al. (129)	Humans: case series	Cannabis	THC:CBD (27:25 mg/ml) as needed + MET and CBT	Self-reported use	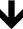
Trigo et al. (123)	Humans	Cannabis	THC:CBD (27:25 mg/ml) as needed + MET and CBT	Abstinence	–
Allsop et al. (130)	Humans	Cannabis	THC:CBD (27:25 mg/ml) + psychosocial intervention	Abstinence	–
Solowij et al. (124)	Humans	Cannabis	Daily oral CBD (200 mg)	Self-reported use	–
Viudez-Martínez et al. (131)	Rats	Ethanol	CBD (30 mg/kg)	Self-administration	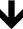
Morgan et al. (125)	Humans	Nicotine	CBD as needed	Self-reported use	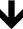
Ren et al. (115)	Rats	Heroin	CBD (5 mg/kg)	Self-administration	–
			CBD (20 mg/kg)	Self-administration	–
Katsidoni et al. (132)	Rats	Morphine	CBD (5 mg/kg)	ICSS threshold	–
		Cocaine	CBD (5 mg/kg)	ICSS threshold	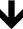
Luján et al. (122)	Mice	Cocaine	CBD (20 mg/kg)	Self-administration	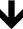
Mahmud et al. (133)	Rats	Cocaine	CBD (5 mg/kg)	Self-administration	–
			CBD (10 mg/kg)	Self-administration	–
Hay et al. (134)	Rats	Methamphetamine	CBD (20 mg/kg)	Self-administration	–
			CBD (40 mg/kg)	Self-administration	–
			CBD (80 mg/kg)	Self-administration	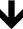
**EXTINCTION**					
Parker et al. (113)	Rats	Cocaine	CBD (5 mg/kg)	CPP	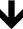
		Amphetamine	CBD (5 mg/kg)	CPP	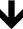
**WITHDRAWAL**					
Crippa et al. (135)	Human: case study	Cannabis	CBD (600 mg)	MWC	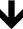
Allsop et al. (130)	Humans	Cannabis	THC:CBD (27:25 mg/ml) + psychosocial intervention	CWS	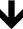
Trigo et al. (123)	Human	Cannabis	THC:CBD (27:25 mg/ml) as needed + MET and CBT	MWC	–
Hindocha et al. (126)	Humans	Nicotine	CBD (800 mg)	MPSS	–
de Carvalho and Takahashi (71)	Rats	Morphine	CBD (10 mg/kg)	CPP following naltrexone-precipitated withdrawal	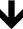
Hine et al. (136)	Rats	Morphine	CBD (10 mg/kg)	Abstinence symptoms	–
			THC:CBD (2:10 mg/kg)	Abstinence symptoms	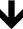
Bhargava (137)	Mice	Morphine	CBD (5 mg/kg)	Naloxone-precipitated withdrawal	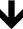
			CBD (10 mg/kg)	Naloxone-precipitated withdrawal	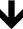
			CBD (20 mg/kg)	Naloxone-precipitated withdrawal	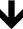
Chesher and Jackson (138)	Rats	Morphine	CBD (5 mg/kg)	Naloxone-precipitated withdrawal	–
			CBD (20 mg/kg)	Naloxone-precipitated withdrawal	–
			CBD (80 mg/kg)	Naloxone-precipitated withdrawal	–
**REINSTATEMENT**					
**Drug-primed**					
Ren et al. (115)	Rats	Heroin	CBD (5–20 mg/kg)	Self-administration	–
de Carvalho and Takahashi (71)	Rats	Morphine	CBD (10 mg/kg)	CPP	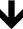
Luján et al. (122)	Mice	Cocaine	CBD (20 mg/kg)	Self-administration	–
Karimi-Haghighi and Haghparast (139)	Rats	Methamphetamine	CBD (10 μg/5 μl)	CPP	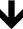
Hay et al. (134)	Rats	Methamphetamine	CBD (20 mg/kg)	Self-administration	–
			CBD (40 mg/kg)	Self-administration	–
			CBD (80 mg/kg)	Self-administration	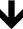
**Context-induced**					
Viudez-Martínez et al. (131)	Rats	Ethanol	CBD (60 mg/kg)	Self-administration	–
			CBD (120 mg/kg)	Self-administration	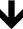
Gonzalez-Cuevas et al. (140)	Rats	Alcohol	CBD (15 mg/kg)	Self-administration	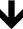
		Cocaine	CBD (15 mg/kg)	Self-administration	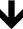
		Cocaine	CBD (10 mg/kg)	CPP	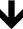
de Carvalho and Takahashi (71)	Rats	Morphine	CBD (5 mg/kg)	CPP	–
			CBD (10 mg/kg)	CPP	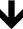
**Cue-induced**					
Ren et al. (115)	Rats	Heroin	CBD (5–20 mg/kg)	Self-administration	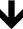
Mahmud et al. (133)	Rats	Cocaine	CBD (5 mg/kg)	Self-administration	–
			CBD (10 mg/kg)	Self-administration	–
**Stress-induced**					
Gonzalez-Cuevas et al. (140)	Rats	Alcohol	CBD (15 mg/kg)	Self-administration	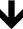
		Cocaine	CBD (15 mg/kg)	Self-administration	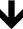

* *CBD, cannabidiol; THC, delta-9-tetrahydrocannabinol; MET, motivational enhancement therapy; CBT, cognitive behavioral therapy; MCQ, marijuana craving questionnaire; CEQ, Cannabis Experiences Questionnaire; TCQ, tiffany craving scale; QSU-B, questionnaire of smoking urges–brief; CPP, conditioned place preference; ICSS, intercranial self-stimulation; MWC, marijuana withdrawal checklist; CWS, cannabis withdrawal scale; MPSS, mood and physical symptoms scale craving*.

### Cannabis

Pharmacological approaches to treating cannabis dependence via agonist replacement (i.e., Dronabinol and Nabilone) have limited efficacy (141). CBD itself has been trialed in rats, and found to be effective in ameliorating conditioned place aversion (CPA) produced by THC injection, but did not alter CPP (142). In human case studies, CBD has also been found to reduce self-reported cannabis use to non-use in a dependent male (128), and to reduced cannabis withdrawal in another (135), although the latter case study did find the subject to have relapsed after a 6-months follow up (135). CBD may have potential in reducing euphoria associated with cannabis use, despite not directly reducing cannabis use (124). However, investigative efforts with pure CBD have been limited. Instead most studies have focused on nabiximols—an oromucosal spray containing 2.7 mg of THC and 2.5 mg of CBD—for cannabis dependence (143).

A number of human case studies suggest nabiximols to be efficacious, in combination with behavioral therapy, in reducing cannabis use and withdrawal symptoms (129). However, case study evidence should be taken cautiously. Further case-control studies indicate nabiximols to be effective in reducing withdrawal, but not cannabis use (123, 130, 144). Nor did it improve abstinence rate (123). It was noted that while therapeutics may assist in short-term withdrawal, it is unlikely that ongoing abstinence can be achieved without psychosocial or clinical support (145). Additionally, the THC component of nabiximols causes the drug to have abuse potential and should not be used lightly (146).

### Alcohol

In animal studies, CBD was effective in reducing ethanol self-administration, and at high enough concentration (120 mg/kg but not 60 mg/kg) attenuated ethanol relapse (131). Further animal studies show CBD (at 15 mg/kg) to effectively reduce cue and stress induced reinstatement of ethanol administration, up to 138 days post-CBD treatment (140). However, one study found CBD alone to be ineffective in attenuating ethanol sensitization, which is suggested to be the first step in drug-associated plasticity (121). Comparatively, pure THC and a 1:1 ratio of THC:CBD was found to be more efficacious in reducing ethanol sensitization. In a human trial of 10-weeks of daily CBD administration in cannabis users, no changes in alcohol or tobacco use was observed either, although the study sample was not dependent on alcohol (124).

### Tobacco

In a placebo controlled study of 24 smokers, those who received a CBD inhaler significantly reduced the number of smoked cigarettes relative to the placebo group, despite no reported difference in craving between groups (125). In another study, oral CBD reduced the salience of cigarette cues, after overnight abstinence in smokers, relative to placebo, but did not reduce craving or withdrawal (126).

### Opioids

Initial studies on the efficacy of cannabinoids in alleviating morphine withdrawal and abstinence symptoms occurred 40 years ago, with rodent models suggesting that CBD alone has low efficacy in alleviating signs of abstinence in rats, but CBD in combination with THC (5:1 ratio) did so significantly (136). THC itself was demonstrated to be more effective than CBD in inhibiting morphine abstinence syndrome in mice (137, 138). Nevertheless, more recent studies demonstrate that treatment with CBD blocked the reward-facilitating effect of morphine (132), reduced morphine CPP and CPA, and prevented drug and stress-induced reinstatement of CPP (71, 127). CBD was also found to have some efficacy in heroin studies in rats. While it did not specifically alter maintenance of self-administration, nor did it aid extinction of self-administration, it did attenuate cue-induced (but not drug-primed) self-administration following 14 days of abstinence, with CBD's effect lasting up to 2 weeks post-administration (115).

### Stimulants

Evidence of CBD efficacy for stimulant use is mixed. Neither CBD, nor a 1:1 ratio of THC:CBD reversed the cocaine sensitization effect (although rimonabant did) (54, 122). Some studies suggest that acute CBD administration does not block the reward-facilitating effect of cocaine (132), reduce cocaine self-administration, or attenuate cue-induced cocaine seeking in rats (133). However, others did find CBD to disrupt acquisition of cocaine self-administration and CPP (122), and impair drug-primed reinstatement of CPP for methamphetamine (139). Further studies on relapse are similarly mixed with one demonstrating CBD's ability to attenuate reconsolidation of CPP (1 week post-CPP acquisition) for cocaine in mice (71), and effectively reduce cue and stress-induced reinstatement of cocaine seeking up to 48 days post-CBD treatment (140), whilst another suggested no effect of CBD on drug-primed reinstatement post-extinction (122). Dose dependency may explain contradictory findings, as Hay et al. (134) demonstrated that 80 mg/kg (and not less) of CBD was needed to significantly reduce motivation to self-administer methamphetamine and reinstatement post-extinction. While evidence for CBD use for stimulant addiction in animals is weak, a longitudinal observational study of 122 participants did find cocaine users who self-report using cannabis to control their cocaine use, to have reduced their cocaine use over a 3 years period (147). Nevertheless, street cannabis generally has low amounts of CBD (148) and findings cannot be extrapolated to CBD's therapeutic efficacy.

The relatively weaker evidence of CBD in disrupting the reward-facilitating effect and self-administration of substances of abuse, despite its comparative efficacy in CPP reinstatement paradigms, may reflect its role in attenuating reward-related memory, without altering the rewarding properties of substances *per se*. Evidence of CBD's role in regulating emotional memory is supported by studies of other conditions, such as anxiety and PTSD-related fear memory [see (47) and (141) for a more extensive review of cannabinoid's role in emotional memory processing across other paradigms]. However, evidence of CBD's role in the consolidation and extinction of substance-related memory in humans is yet limited.

## Summary and Future Directions

CBD shows some promise in alleviating negative withdrawal effects and reducing motivation to self-administer or reinstatement of drug use in animals. However, evidence on its efficacy is limited and mixed. CBD alone may not be sufficiently effective in maintaining long-term abstinence without ongoing support and behavioral therapy, as evidenced by its lack of efficacy over treatments, such as cognitive behavioral therapy and motivational enhancement therapy (123, 129). A combination of pharmacotherapy and behavioral therapy may increase treatment potency and adherence (149), and CBD may be better suited as an adjunct treatment to primary behavioral or psychosocial therapy (124).

There is also much that is unknown about how CBD may be targeting and alleviating SUD-related effects. Recent evidence suggests that within the mesolimbic system, CBD also influences the serotonergic system, as an agonist of the serotonin 1A (5-HT_1A_) receptor (102, 103), which in addition to contributing to reduction in stress and anxiety (150), may be responsible for (i) blunting the reward-facilitating effect of substances of abuse (e.g., morphine in rats) (132); and (ii) modulating the formation of associative emotional memory related to substances of abuse (151). A number of studies have suggested the potential of selective serotonin reuptake inhibitors and other antidepressants in reducing substance (e.g., alcohol and nicotine) use via alleviating mood symptoms (152). CBD's capacity to alleviate stress, anxiety, and depressive symptoms may be mediating its treatment effect on SUDs (124, 153, 154). Indeed, CBD has been found to have therapeutic potential in alleviating affective and cognitive processing disturbances that may be induced by chronic substance (e.g., cannabis) use (63, 64, 155), proving potential utility in moderating the deleterious course of impairment, particularly in adolescent initiates of substance use (156). Additionally, other receptor and enzyme functions targeted by CBD, such as cannabinoid CB2Rs, non-cannabinoid transient receptor potential vanilloid type-1 (TRPV1) and type-2 (TRPV2) receptors, and ECS' catabolic enzymes FAAH and MAGL, should also be investigated for their role in the ECS and SUD (157–161).

In sum, some early research supports CBD's promise as pharmacotherapy against SUD. However, further investigation into CBD's mechanism of action, and validation of its efficacy, across preclinical and clinical trials will be necessary.

## Author Contributions

YC wrote the core sections of the manuscript with the assistance of EC. NS and MY provided intellectual input and edits.

### Conflict of Interest Statement

The authors declare that the research was conducted in the absence of any commercial or financial relationships that could be construed as a potential conflict of interest.
